# Investigation of the Selectivity of L-Type Voltage-Gated Calcium Channels 1.3 for Pyrimidine-2,4,6-Triones Derivatives Based on Molecular Dynamics Simulation

**DOI:** 10.3390/molecules25225440

**Published:** 2020-11-20

**Authors:** Qi Ye, Zhenyu Zhang, Wenying Zhang, Yushan Ding, Fan Zhao, Jinghai Zhang, Yongbo Song

**Affiliations:** School of Life Science and Biopharmaceutics, Shenyang Pharmaceutical University, 103 Wenhua Road, Shenyang 110016, China; yeokqi@163.com (Q.Y.); zzy18306510590@163.com (Z.Z.); wy2549925684@foxmail.com (W.Z.); dinng814@163.com (Y.D.); syphurabbit@163.com (F.Z.); zhangjinghai@syphu.edu.cn (J.Z.)

**Keywords:** hCa_v_1.2, hCa_v_1.3, selective inhibitor, pyrimidine-2,4,6-triones, molecular dynamics simulation

## Abstract

Human Ca_v_1.3 (hCa_v_1.3) is of great interest as a potential target for Parkinson’s disease. However, common medications like dihydropyridines (DHPs), a kind of classic calcium channel blocker, have poor selectivity to hCa_v_1.3 in clinical treatment, mainly due to being implicated in cardiovascular side-effects mediated by human Ca_v_1.2 (hCa_v_1.2). Recently, pyrimidine-2,4,6-triones (PYTs) have received extensive attention as prominent selective inhibitors to hCa_v_1.3. In this study, we describe the selectivity mechanism of PYTs for hCa_v_1.2 and hCa_v_1.3 based on molecular dynamic simulation methods. Our results reveal that the van der Waals (vdW) interaction was the most important force affecting selectivity. Moreover, the hydrophobic interaction was more conducive to the combination. The highly hydrophobic amino acid residues on hCa_v_1.3, such as V162 (IR1), L303 (IR2), M481 (IR3), and F484 (IR3), provided the greatest contributions in the binding free energy. On the other hand, the substituents of a halogen-substituted aromatic ring, cycloalkyl and norbornyl on PYTs, which are pertinent to the steric hindrance of the compounds, played core roles in the selectivity and affinity for hCa_v_1.3, whereas strong polar substituents needed to be avoided. The findings could provide valuable information for designing more effective and safe medicines for Parkinson’s disease.

## 1. Introduction

Voltage-gated calcium channels (VGCCs) are important sensors that can convert changes in cell surface membrane potential into intracellular physiological activity changes [1]. Allowed to go through the central pore region to enter or release to the sides of the cell membrane, calcium ions could regulate intracellular calcium ion concentration [2]. Calcium ions in the cell act as second messengers for activating a range of physiological activities such as muscle contraction, neurotransmitter release, hormone secretion and gene expression [3]. According to electrophysiological standards, VGCC can be divided into high voltage-activation (HVA) types and low voltage-activation (LVA) types. The HVA is consisted of the L-type (Ca_v_1 family), R-, P-/Q-, and N-type (Ca_v_2 family), while the LVA is only composed of the T-type (Ca_v_3 family) [4,5]. The members of the Ca_v_1 subfamily are classified into four isoforms (Ca_v_1.1 to Ca_v_1.4) according to their different structures and expression distributions in physiological functions [5,6]. L-type voltage-gated calcium channels (LTCCs) are heteropolymers consisting of α1, α2, β, γ and δ subunits [7]. α1 is crucial and can determine most of the biophysical and pharmacological properties of LTCCs. This subunit consists of four highly homologous transmembrane domains (DI-IV). Each domain contains six transmembrane spirals (S1–S6). Among them, S1–S4 form the voltage sensor domains (VSD), and S5–S6 with the extracellular regions (P-loops) constitute the central pore region of the channel [7,8]. Expressed in heart, smooth muscle, pancreas and brain, Ca_v_1.2 is the major and most widespread isoform (about 90%) in the LTCCs. However, Ca_v_1.3 mainly remains in the neuron system [9,10]. This specificity of tissue distribution has led to many diseases closely related to LTCCs [11,12]. For example, human Ca_v_1.2 (hCa_v_1.2) is one of the important targets of cardiovascular diseases, whereas human Ca_v_1.3 (hCa_v_1.3) is involved in the pathogenesis of Parkinson’s disease (PD) and is considered to be a therapeutic target for neurodegenerative diseases [13,14,15,16,17].

Several research reports have indicated some promising results for the traditional LTCCs’ antagonists, such as the dihydropyridines (DHPs), benzothiazepines (BTZs) and phenylalkylamine (PAAs). Particular interest to the class of DHPs made them widely used in clinical treatment so that LTCCs are also termed as dihydropyridines receptors [18,19,20,21,22,23,24]. Due to the lack of selectivity of available pharmacological agents for hCa_v_1.2 and hCa_v_1.3, present medicines like DHPs for the treatment of neurodegenerative diseases are likely to cause potential cardiovascular side-effects mediated by hCa_v_1.2 [2]. Koscha et al. indicated that isradipine had a similar affinity to hCa_v_1.2 and hCa_v_1.3, but its inhibition rate of hCa_v_1.2 was about 10-fold higher than that of hCa_v_1.3 due to the difference in voltage dependence [25].

Among these studied compounds, none indicated high selectivity for hCa_v_1.3 [10,25,26,27,28] except some pyrimidine-2,4,6-triones (PYTs) reported recently by Kang et al. In their research, the PYTs’ scaffold was identified as the first class of selective antagonists for hCa_v_1.3. Especially, the compound (1-(3-chlorophenethyl)-3-cyclopentylpyrimidine-2,4,6-trione) exhibited much higher IC_50_ of hCa_v_1.3 (1.7 μM) in contrast to hCa_v_1.2 (1162 μM) [29]. Furthermore, many other PYTs have also been tested for their selectivity to hCa_v_1.3 and hCa_v_1.2 and the results are similar to this compound [30]. It can be seen that PYTs could be a potential selective antagonist for hCa_v_1.3. In addition, the active pocket that binding to benzodiazepines on LTCC is also reported as the binding site to PYTs [31]. These provide experimental support for elucidating the selective mechanism of these compounds to hCa_v_1.2 and hCa_v_1.3. However, limited by the complex structures of LTCCs, exploring the interactions mentioned above relying on experimental methods consumes a lot of resources. Bioinformatics, which has been widely recognized and applied, becomes the best choice to solve this issue. In 2014, publication of the crystal structure of the calcium channel from the gram-negative pathogen *Arcobacter butzleri* (Ca_v_Ab) paved the way for an in-depth understanding of LTCCs [32]. Recently, the structure solution of rabbit Ca_v_1.1 (rCa_v_1.1) through cryoelectron microscopy strengthened the foundation of research on calcium channel structure and function [33].

**Figure 1:**
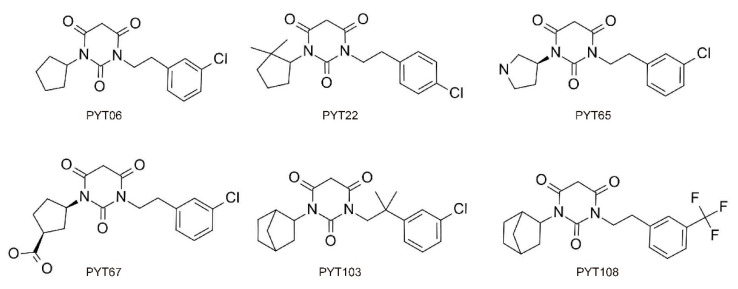
The chemical structure of selected pyrimidine-2,4,6-triones (PYTs) [30].

In this study, computational simulation methods were applied to investigate the differences in the selectivity of PYTs to hCa_v_1.2 and hCa_v_1.3. Based on the crystal structure of rCa_v_1.1, the pore domains of hCa_v_1.2 and hCa_v_1.3 were built by computer dynamics simulation. Two groups of PYTs (group A: PYT06, PYT22, PYT65; group B: PYT67, PYT103, PYT108; Figure 1 and Table S1 in Supplementary Material) were selected from previous studies [30]. The three compounds in each group were selected with high, low and no inhibition rates to hCa_v_1.3 and hCa_v_1.2. The specific binding characteristics and related key factors are described in detail to clarify the mechanism of hCa_v_1.3 selectivity. Our study could provide theoretical support and ideas for future studies of selective inhibitors of PYTs as well as the selective inhibitors of LTCCs.

## 2. Results

### 2.1. Analysis of 3D Structures of Pore Domains on hCa_v_1.3 and hCa_v_1.2

**Figure 2:**
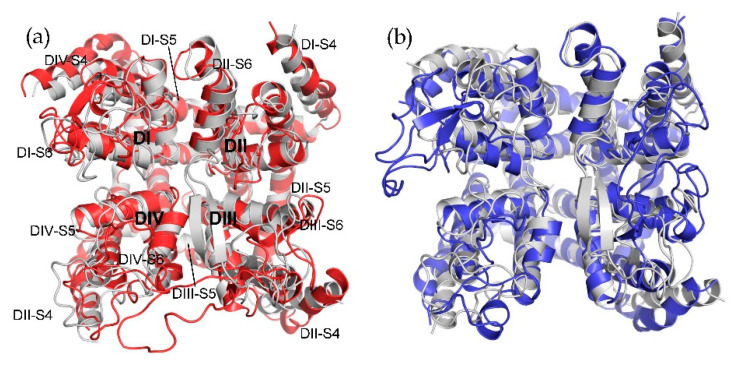
The structure alignment between the initial structure (gray) and simulated structure (red and blue) for hCa_v_1.3 (**a**) and hCa_v_1.2 (**b**). The domain was marked in hCav1.3 based on the names.

The sequence identity of pore domains on hCa_v_1.3/Ca_v_1.2 and rCa_v_1.1 was up to 70% (Figure S1 in Supplementary Material), which ensured the accuracy of these two models to a certain extent. As mentioned above, a total of 500 homology models were established, and the best models were selected based on the DOPE value and Molpdf score according to their energy [34]. The structures of hCav1.3/Cav1.2 (Figure S2 in supplementary material) were evaluated by ERRAT, PROCHECK, and WHATCHECK [35,36,37,38], and the results are shown in Supplementary Table S2. The average values of the overall quality factors of ERRAT are 70.75 and 73.89, respectively, showing a great quality for two structures. The Ramachandran plot in PROCHECK was used to analyze residue-by-residue geometry and overall structure geometry of the protein structure. The analysis results (Figure S3 in Supplementary Material) indicated that about 98.5% and 97.5% residues in hCa_v_1.3 and hCa_v_1.2, respectively, were in the most favored regions and additional allowed regions. Moreover, the residues in the disallowed regions were generally the terminal residues. The root-mean-square Z-scores (RMS Z-scores) of bond lengths and bond angle from the WHATCHECK also confirmed that the model had better stereochemical properties compared to other models. Additionally, 50 ns preliminary simulation was performed on the initial structure to verify stability. The structure alignment between the initial structure and simulated structure of hCa_v_1.3 and hCa_v_1.2 returned the root-mean-square-deviations (RMSD) of 2.3 Å and 2.5 Å (Figure 2), respectively. It was shown that hCa_v_1.2 was more unstable than hCa_v_1.3 and the main fluctuant regions of hCa_v_1.2 were located in DI and DIII. Few displacements were detected in the transmembrane helices near the binding site for both. Subsequently, the homology models of hCa_v_1.3 and hCa_v_1.2 were prepared for membrane-embedded protein simulation with water as solvent (Figure S4 in Supplementary Material) for further studies.

### 2.2. Molecular Dynamics Simulation of hCa_v_1.3/Ca_v_1.2-PYTs Complexes

**Figure 3:**
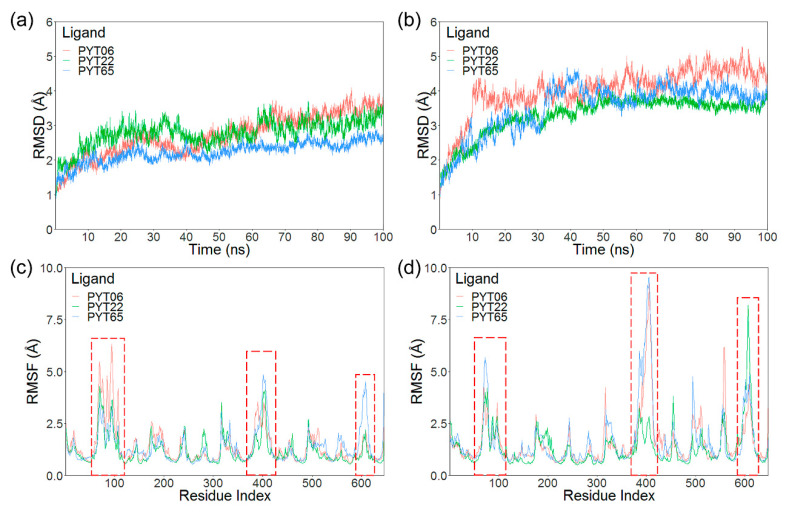
RMSD curves of the systems of hCa_v_1.3 (**a**) and hCa_v_1.2 (**b**) with PYT06 (pink), PYT22 (green), and PYT65 (blue). RMSF curves by residues in hCa_v_1.3 (**c**) and hCa_v_1.2 (**d**) systems. The main fluctuating regions were enclosed in red dashed rectangles.

Molecular docking was used to generate the initial structures of molecular dynamics (MD) simulation, and the best complexes of hCa_v_1.3/hCa_v_1.2 were selected based on the docking conformation and binding energy. MD simulations of 100 ns were carried out to the hCa_v_1.3/Ca_v_1.2-PYTs complexes. The RMSD of hCa_v_1.3 and hCa_v_1.2 for PYTs in group A (PYT06, PYT22, and PYT65) are exhibited in Figure 3a,b. It was shown that the last 40 ns were relatively stable for all complex systems. However, the systems related to hCa_v_1.2 obviously had a larger average RMSD value (4.02 Å) compared with the systems related to hCa_v_1.3 (2.94 Å), especially the complex of hCa_v_1.2-PYT06 (red curve in Figure 3b) whose average RMSD value was 4.48 Å in the final 40 ns, exhibiting a greater range of fluctuation than the other complexes. In consideration of the same template used for constructing the models of hCa_v_1.2 and hCa_v_1.3, it is reasonable to believe that these differences were generated by different affinities of binding ligands.

As shown in Figure 3c,d, the root-mean-square fluctuation (RMSF) curves indicated that three main fluctuating regions (marked with red dashed rectangles in Figure 3) in these two LTCCs were found. These regions were mainly located in the extracellular loops between S5 and S6. Except for flexible regions in the terminals of the receptors, most of the residues showed few movements in the three complexes of hCa_v_1.2. Besides, it was noticed that the second fluctuating region (383th–416th residues) in the complexes of hCa_v_1.2-PYT06 and hCa_v_1.2-PYT65 exhibited stronger fluctuations than the others.

Each hCa_v_1.3 and hCa_v_1.2 complex system eventually became steady during the whole simulation. However, the fluctuation of hCa_v_1.2 complex systems was greater than that of the hCa_v_1.3 complex systems, which was particularly obvious in hCa_v_1.2-PYT06/PYT65 complexes. According to the experimental data [29], the PYT06 and PYT65 had low inhibition on hCa_v_1.2. Therefore, it seemed that the low inhibition of the ligand could result in structural instability for hCa_v_1.2, but no such situation was found in hCa_v_1.3 complex systems. Thus, hCa_v_1.2 seemed to be more sensitive to the binding strength of the ligand.

### 2.3. Analysis of Binding Modes of Complex Systems

The molecular mechanics/generalized Born surface area (MM/GBSA) method was carried out in the last 20 ns of MD trajectories. As can be seen in Table 1, the total free binding energy of each complex was calculated and the results were generally consistent with the selectivity of experimental data. Compared to hCa_v_1.2, PYT06 had a high selectivity to hCa_v_1.3, PYT22 had strong binding affinities for both, whereas PYT65 was weak in combination with both. Moreover, the van der Waals interaction energy (ΔE_vdW_) was confirmed as the primary influence factor of the binding free energy in the binding of PYTs with hCa_v_1.2 and hCa_v_1.3.

**Figure 4:**
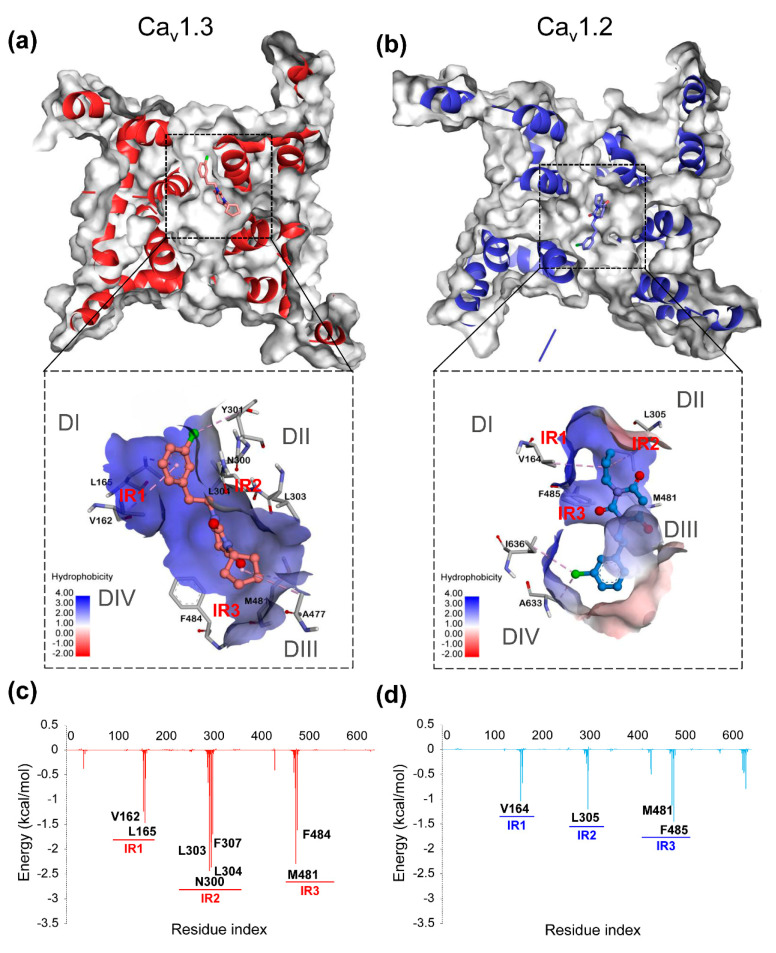
The binding conformation with the hydrophobic surface of the complexes of hCa_v_1.3-PYT06 (**a**) and hCa_v_1.2-PYT06 (**b**). Deeper blue area on the surface indicates high hydrophobicity and vice versa. The energy decomposition of complexes of hCa_v_1.3-PYT06 (**c**) and hCa_v_1.2-PYT06 (**d**). The main interaction regions (IR) are marked in the figure.

Furthermore, 3D binding conformations and energy decomposition of per-residue were used to determine the critical residues and regions in the combinations of PYTs to hCa_v_1.2 and hCa_v_1.3. The major interactional regions (termed IR1-IR4) in four domains of each system are listed in Supplementary Table S3, where residues with high energy contributions are shown. As shown in Figure 4, in the system of hCa_v_1.3-PYT06, three major interaction regions (IR1-IR3) were obviously found in the binding pocket located in DI, DII, and DIII, respectively. Among them, IR2 had the highest energy contribution to the binding free energy, and the binding site in the center pore remained highly hydrophobic. The PYT06 formed Pi-Alkyl hydrophobic contacts with V162 and L165 on hCa_v_1.3 through chlorophenyl and interacted with N300, L304, and M481 as well. These important residues with other hydrophobic residues formed an intensive hydrophobic surface around the binding pocket. However, in comparison to active pockets on hCa_v_1.2 and hCa_v_1.3 when combined with PYT06, there was only residue L305 in the IR2 in hCa_v_1.2. Moreover, the interactive regions showed a reduction of residue energy contribution (Figure 4c,d) for the hCa_v_1.2-PYT06 complex, although it had similar interactive regions to the hCa_v_1.3-PYT06 complex. The binding site of hCa_v_1.2 and PYT06 was closer to DIII and DIV, and this region had lower hydrophobicity. Therefore, compared with hCa_v_1.2, PYT06 had the strongest selectivity for hCa_v_1.3 because it had higher hydrophobic interaction.

**Figure 5:**
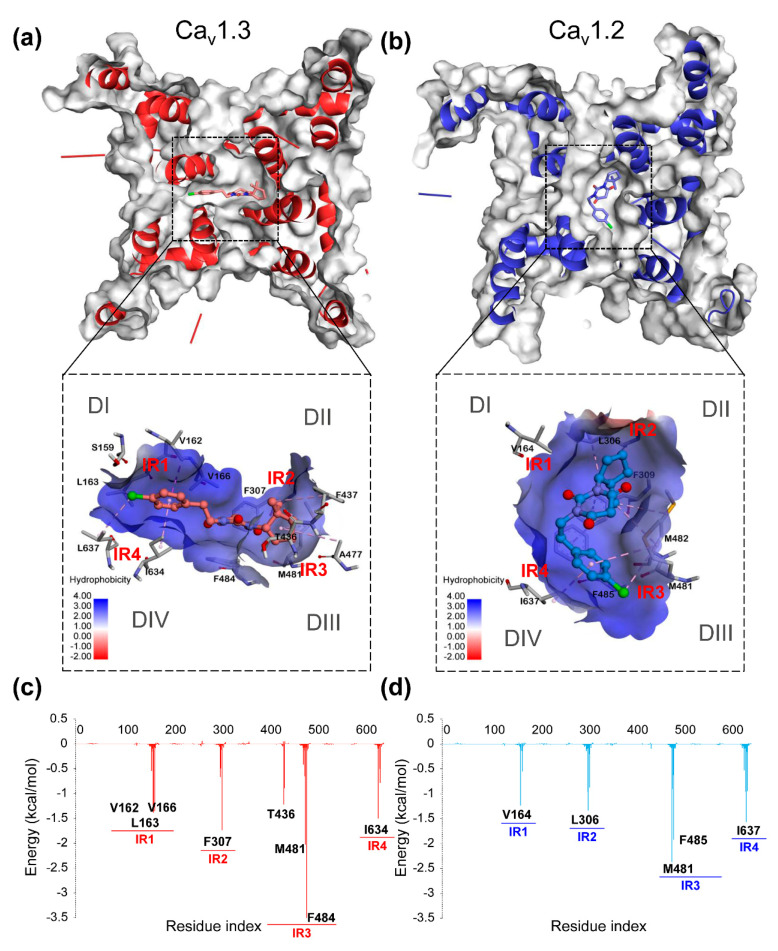
The binding conformation with the hydrophobic surface of the complexes of hCa_v_1.3-PYT22 (**a**) and hCa_v_1.2-PYT22 (**b**). Deeper blue area on the surface indicates high hydrophobicity and vice versa. The energy decomposition of complexes of hCa_v_1.3-PYT22 (**c**) and hCa_v_1.2-PYT22 (**d**). The main interaction regions (IR) are marked in the figure.

As can be seen in Figure 5, for complexes of hCa_v_1.2-PYT22 and hCa_v_1.3-PYT22, the regions with great energy contributions could be divided into four parts (IR1-IR4), where the locations were all similar. Interestingly, most of the residues in these four regions were hydrophobic amino acids. The low binding free energy was both reflected in the complexes of hCa_v_1.2-PYT22 (−29.6581 kcal/mol) and hCa_v_1.3-PYT22 (−35.1939 kcal/mol). On the one hand, M481 and F484 of hCa_v_1.3, M481, and F485 of hCa_v_1.2 in IR3 contributed the most to the combination. On the other hand, the chlorophenyl on PYT22 formed the Pi-alkyl interactions and alkyl (-Cl) interactions with V162/I634 and L163/L637 on hCa_v_1.3, respectively. For the hCa_v_1.2, there were also the Pi-alkyl interactions and alkyl (-Cl) interactions between the chlorophenyl on PYT22 and M481/I637 and Met481. The distinction of residues in IR1 between hCa_v_1.3 (V162, L163, V166) and hCa_v_1.2 (V164) was the main reason that caused different affinity of PYT22 to both. The dimethyl on the cyclopentyl ring of PYT22 provided more potential for binding free energy. Thus, the binding free energy of the two complexes that bound PYT22 was relatively low, which was consistent with the experiment results.

**Figure 6:**
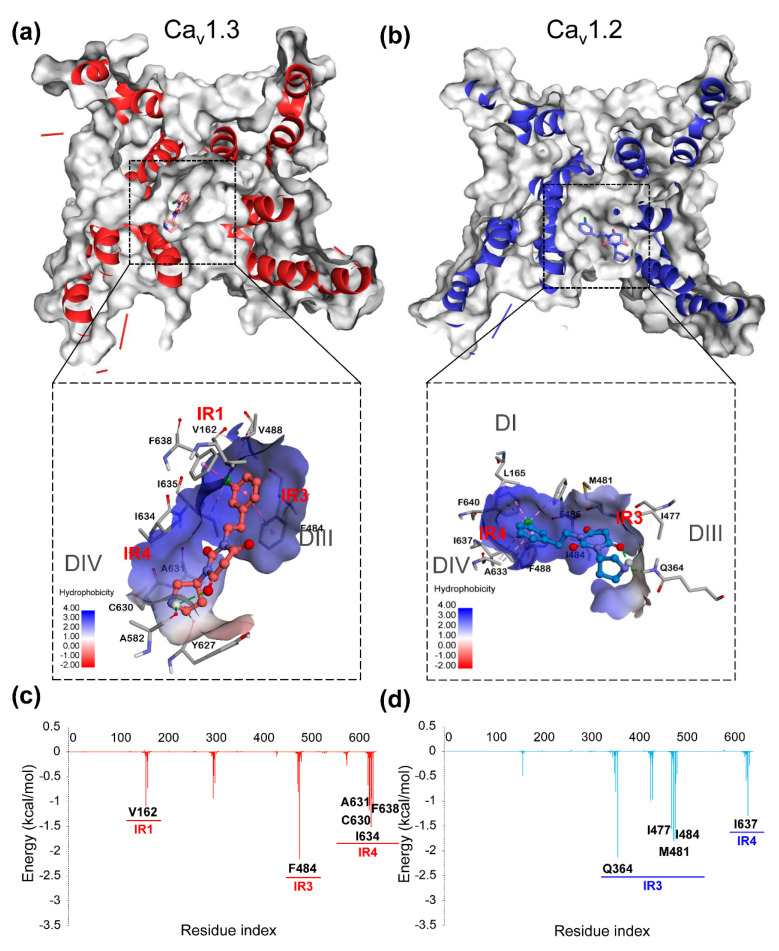
The binding conformation with the hydrophobic surface of the complexes of hCa_v_1.3-PYT65 (**a**) and hCa_v_1.2-PYT65 (**b**). Deeper blue area on surface indicates high hydrophobicity and vice versa. The energy decomposition of complexes of hCa_v_1.3-PYT65 (**c**) and hCa_v_1.2-PYT65 (**d**). The main interaction regions (IR) are marked in the figure.

As shown in Figure 6, in contrast to PYT22, PYT65 displayed a large difference when combined with hCa_v_1.2 and hCa_v_1.3. Because of the polar nitrogen of pyrrolidine, PYT65 bound to the less hydrophobic pocket that was closer to DIII and DIV on the receptors. This led to critical interaction residues concentrated on IR3 and IR4. Meanwhile, the binding site of PYT65 deviated from the center pore region, which caused reduction of hydrophobic interactions. The contributions made by the residues on DI and DII were significantly reduced, especially on hCa_v_1.2. It could be deduced that this was the main reason that caused the complexes with PYT65 to show higher binding free energy than the complexes of PYT22.

As a whole, it was not difficult to find that the highly hydrophobic surface made by residues in IR2 and IR3 was the major factor to influence the selectivity to Ca_v_1.3. The nonpolar substituent on the PYT ring could improve the interaction of PYTs with hCa_v_1.3/Ca_v_1.2.

### 2.4. Verification of the Binding Mode

The duplicate process was carried out with the same parameter for each system in group A, and the binding free energy and per-residue energy decomposition were calculated. The results of the binding free energy are listed in Supplementary Table S2. Compared to hCa_v_1.2, it is not surprising that the PYT108 showed a higher affinity to hCa_v_1.3, which was consistent with experimental data. For the PYT103 with the high affinity to hCa_v_1.2 and hCa_v_1.3, both the hCa_v_1.2-PYT103 and hCa_v_1.3-PYT103 had low binding free energy, and the hCa_v_1.3-PYT67 and hCa_v_1.2-PYT67 showed much higher values of binding free energy.

**Figure 7:**
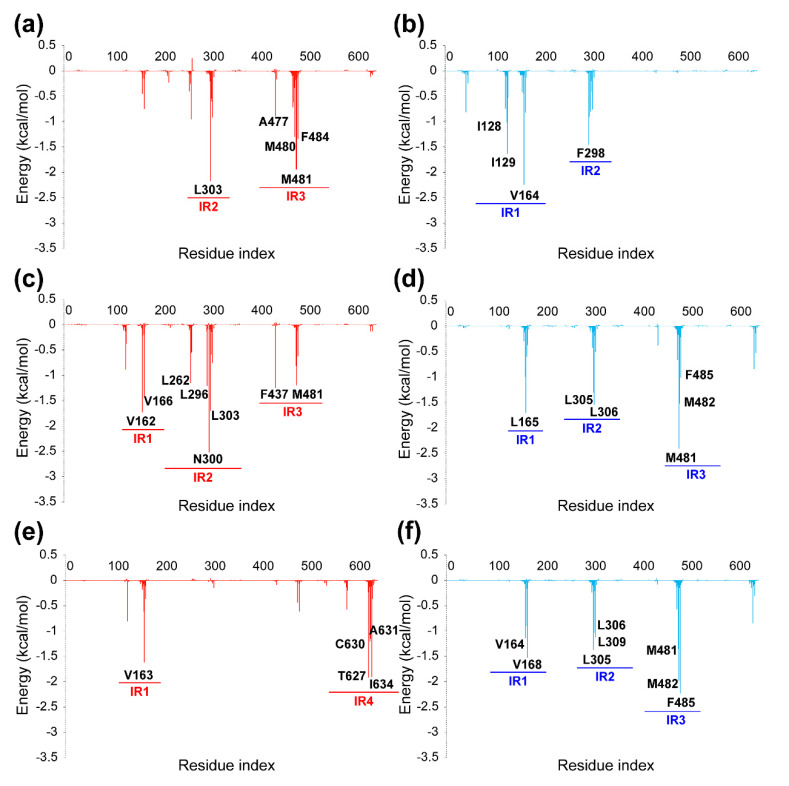
The per-residue energy decomposition of the complexes of PYT108 (**a**,**b**) and PYT103 (**c**,**d**) and PYT67 (**e**,**f**) for hCa_v_1.3 (**a**,**c**,**e**) and hCa_v_1.2 (**b**,**d**,**f**). The main interaction regions (IR) are marked under the corresponding residues.

**Figure 8:**
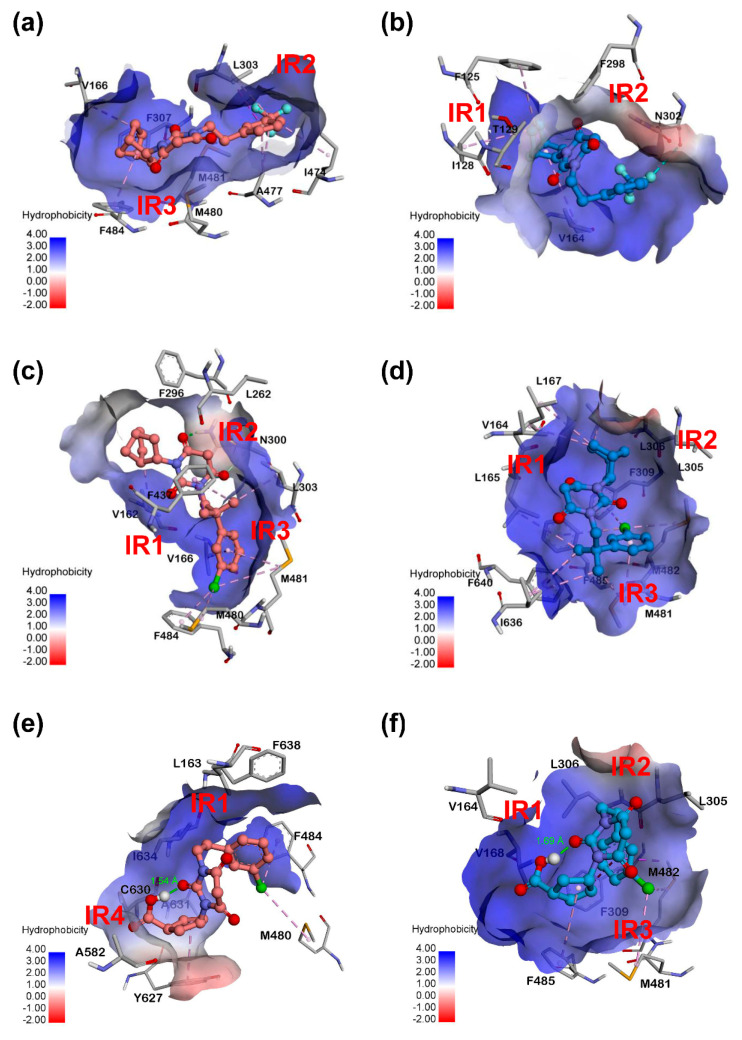
The binding mode with hydrophobic surface of complexes of PYT108 (**a**,**b**), PYT103 (**c**,**d**), and PYT67 (**e**,**f**) for hCa_v_1.3 (**a**,**c**,**e**) and hCa_v_1.2 (**b**,**d**,**f**). The main interaction regions (IR) are marked in red.

In order to further confirm the key factors that affect the selectivity difference of PYTs on the hCa_v_1.3/Ca_v_1.2, other PYTs (group B) were selected to verify the results obtained by group A again. As displayed in Figure 7 and Figure 8, the energy decomposition of residues and the structures with the hydrophobic surface provided valuable information for the selectivity of hCa_v_1.3/Ca_v_1.2. The residues in each of the main interactive regions of hCa_v_1.3 and hCa_v_1.2 complexes are listed in Supplementary Table S4. In the hCa_v_1.3-PYT108, the residues in IR2 and IR3 were found to be the main interactive regions to retain the high binding free energy. The residues L303 in IR2, M480, and M481 in IR3 played an important role in the energy contribution of hCa_v_1.3-PYT108. It was also confirmed in the hCa_v_1.3-PYT06 system that these residues were more important to the selectivity of hCa_v_1.3. In contrast, the hCa_v_1.2-PYT108 could not retain the low binding free energy due to the lack of residues with high energy contribution in IR2 and IR3.

According to Figure 7c,d, PYT103 had a similar high energy contribution to hCa_v_1.3 and hCa_v_1.2. The residues in IR1, IR2, and IR3 of hCa_v_1.3 and hCa_v_1.2 formed interaction with the ligand. Besides, it was found in the binding mode (Figure 8a–d) that the norbornyl groups in PYT108 and PYT103 helped to form hydrophobic interactions with the surrounding amino acid residues. As shown in Figure 7e,f, the decreased energy contribution was shown in hCa_v_1.3-PYT67 and the main interaction residues were in the IR4. Compare with the hCa_v_1.3-PYT108 or hCa_v_1.3-PYT103 system, it indicated that IR2 and IR3 were requisite for high binding affinity. It was confusing, however, that the hCa_v_1.2-PYT67 system showed high contribution of residues. These were considered to be related to the structure of PYT67 itself. The binding mode showed that the intramolecular hydrogen bond was formed in the PYT67 for both systems (Figure 8e,f). Therefore, it was inferred that the intramolecular hydrogen bond resulted in the low energy change of the ligand, which seemed to be unfavorable to the binding of PYTs. In summary, the verification of group B further confirmed the difference of binding mode between hCa_v_1.3 and hCa_v_1.2 with PYTs. The spatial structure and polarity of PYTs could affect significantly the binding affinity, and suitable substituents could increase the selectivity to hCa_v_1.3.

## 3. Discussion

hCa_v_1.2 and hCa_v_1.3 are the main isoforms of LTCCs with the widest distribution and expression. However, due to their highly-homologous sequences, similar structures, and pharmacological properties, traditional calcium channel blockers (CCB) used in clinical treatment exhibited poor selectivity to these two subtypes. Based on the experimental data published, PYTs are a kind of potential selective inhibitor to hCa_v_1.3 [29,30]. Therefore, it is necessary to elucidate the mechanism of selectivity of hCa_v_1.3 and hCa_v_1.2.

In this study, six PYTs were divided into two groups (A, B) according to different selectivity and structure. There were two substituents on the PYT ring. One of the substituents was a five-membered ring, such as cyclopentyl (PYT06), dimethylcyclopentyl (PYT22), or pyrrolidinyl (PYT65) in group A, and formyloxycyclopentyl (PYT69) or norbornyl (PYT103, PYT108) in group B. The other one was a halogen-substituted aromatic ring, including trifluoromethylphenyl (PYT108) or chlorophenyl (others).

According to the results of this study, the RMSD and RMSF of the PYTs-hCa_v_1.2/hCa_v_1.3 complex were stable in the central pore region of the transmembrane helix during MD simulation, and the major fluctuations were caused by the extracellular loop. It is worth noting that ligands with low affinity could more affect the overall stability of the receptor hCa_v_1.3/Ca_v_1.2. The binding free energy calculation and conformation analysis showed that hCa_v_1.3-PYT06 and hCa_v_1.3-PYT108 had lower binding free energy than hCa_v_1.2-PYT06 and hCa_v_1.2-PYT108, which was consistent with experimental data and other simulation systems [29]. The components of binding energy suggested that the ΔE_vdW_ term was the main energy contribution to the binding free energy for each system. The complex systems with high affinity retained the low ΔE_vdW_ in general. Moreover, the residues, such as V162, L303, M481, and F484 (residues in hCa_v_1.3), in the binding site contributed most of the binding free energy based on the energy decomposition per-residue. Comparing the binding mode of hCa_v_1.2 and hCa_v_1.3, the difference was mainly attributed to the residues that formed the hydrophobic surface in the IR2 and IR3. In our study, we also found that these differences played a decisive role in the selectivity of hCa_v_1.3/Ca_v_1.2 with different ligands.

As expected, the halogen-substituted aromatic ring in PYTs could contribute the most binding affinity by the formation of hydrophobic interactions. On the other side of the PYT ring, the structure of the substituent played an important role in selectivity. It seemed that cycloalkyl and norbornyl provided the higher selectivity for hCa_v_1.3. The strong polarity group may be not a good choice because it could move the ligand away from the hydrophobic surface, resulting in poor binding affinity and low selectivity, just like the PYT65 and PYT67. Somewhat confusingly, the PYT67 had a poor binding affinity for hCa_v_1.2. It seemed that the carboxyl of PYT67 formed an intramolecular hydrogen bond, which led to the reduction of the energy change itself. Besides, steric hindrance in the binding site limited the interaction of the receptor for the ligand. Therefore, matched space size and a suitable substituent group of PYTs may increase the affinity to LTCCs.

Some of the PYTs were reported as highly selective antagonists of hCa_v_1.3, which could decrease side-effects on the body effectively. However, studies on the mechanism of action and pharmacology of PYTs are deficient somehow. It has been reported that the inhibitory activity of the PYT06 to LTCCs was confirmed, but the selective activity against hCa_v_1.3 was dependent on the β-subunit [39]. Another study indicated the PYTs may be a new class of activator for LTCCs, and the PYT06 could cause a slowing of the activation and inactivation time course, which showed that the selective inhibitory activity to hCa_v_1.3 of PYTs was closely dependent on the experimental conditions [40]. Therefore, further studies of PYTs and other hCa_v_1.3 selective inhibitors need to be performed. We expect that our study could reveal the binding mode of the hCa_v_1.2 and hCa_v_1.3 with the PYTs, which will help research on related disease and drugs targeted to LTCCs in the near future.

## 4. Materials and Methods

### 4.1. Homology Modeling

Homology modeling was applied to build the theoretical models of the α1-subunits on L-type calcium Channels hCa_v_1.2 and hCa_v_1.3. The amino acid sequences of the α1-subunits on hCa_v_1.2 (access number: Q13936) and hCa_v_1.3 (access number: Q01668) were originated from the UniProt database (www.uniprot.org). Sequence alignments of hCa_v_1.2 and hCa_v_1.3 with the sequence of rCa_v_1.1 were carried out by applying ClustalX 2.1 [41]. The rCa_v_1.1 (PDB ID: 5GJV) was selected as the best template to model the hCa_v_1.2 and hCa_v_1.3 α1-subunits. Modeller 9.9 [42] was used to generate the 3D models of the target sequences and the best ones were chosen according to DOPE value and Molpdf score [34]. According to previous reports, the central pore region could retain great credibility [43], and the binding site of PYTs is also located in the pore region. Therefore, the central pore region (S4-S6 composition) of hCa_v_1.2 and hCa_v_1.3 was selected for follow-up detailed analysis, and the optimized structures were submitted to SAVES v5.0 to evaluate their reasonability through ERRAT, PROCHECK, and WHATCHECK [35,36,37,38]. Among them, ERRAT generated the overall quality factor based on the quality of the protein, PROCHECK analyzed the residue-by-residue geometry and WHATCHECK analyzed the great stereochemical parameters via a comprehensive checking for the residues. Considering the real physiological environment, these structures were embedded in a palmitoyl-oleoyl-phosphatidyl-choline (POPC) lipid bilayer using the CHARMM-GUI online tool. Meanwhile, a total of 20952 TIP3P water models were introduced to solvate the system [44].

### 4.2. Molecular Docking of PYTs to hCa_v_1.3 and hCa_v_1.2

Autodock 4.2 [45] was used to predict the docking conformation for PYT at hCa_v_1.3 and hCa_v_1.2. The grid box was defined as a cubic binding pocket with 40 points for every side in the grid spacing of 0.375 Å. Two hundred binding poses were calculated through the genetic algorithm with 2.5 × 10^7^ maximum number of evals and 150 population size, and the default parameters were used for other options. The binding pose of the protein-ligand complexes with the best binding mode and binding free energy was selected by clustering analysis as an initial structure for further molecular dynamics simulations.

### 4.3. Molecular Dynamics Simulations

The AMBER 16 package [46] was used to carry out molecular dynamics (MD) simulations with AMBER-ff14SB force field [47] for the selected complexes, meanwhile, the general AMBER force field (GAFF) [48] and the AMBER Lipid14 force field [49] were employed for the ligands and the POPC lipid bilayer, respectively. The receptor-ligand complex systems with membranes and water molecules were appended to a cubie cell with periodic boundary conditions, while counterions (Cl-) were added to maintain the electrical neutrality of the overall system. The particle-mesh Ewald (PME) algorithm [50] was utilized for energy minimization and molecular dynamics (MD) to calculate the long-range electrostatic interactions with a cutoff value of 12 Å in the whole simulation process. The SHAKE algorithm was applied to constrain all the covalent bonds with hydrogens [51] and Langevin dynamics [52] with a collision frequency γ of 1.0 used to control the change of temperature during MD simulation. Before the MD simulation began, energy minimization was performed in six cycles with a restraining force from 100.0 to 0 kcal/(mol·Å^2^). In each cycle, 2500 steps conjugated gradient minimization and 2500 steps steepest-descent minimization were successively carried out to remove unnatural contact in the system. Then, the whole systems were heated to 310.0 K gradually with solutes restrained using a harmonic potential in the NVT ensemble. 400 ps of NTP simulations were subsequently performed for equilibrations via two steps [53,54]. A decreasing restraining force was applied to solutes gradually in the first step and the second step was carried out without any restraining force. Further, the production MD simulations of 100 ns were run at 1.0 atm and 310.0 K with 2.0 fs time step. All complex systems were processed under the same conditions. The root mean square deviation (RMSD) of backbone atoms and the root mean square fluctuation (RMSF) of residue were calculated by the trajectory analysis tool CPPTRAJ [55] to manifest the stability of complex systems. All the calculations were submitted with the trajectory of a stable period.

### 4.4. Binding Energy Calculations and Decomposition by MM/GBSA

The molecular mechanics/generalized Born surface area (MM/GBSA) method was applied in analyzing the molecular interaction between ligand and receptor [56]. For the MM/GBSA, the ΔGbind (G_Complex_-G_Receptor_-G_Ligand_) were the energy differences, which were broken up into four basic objects, obtaining the van der Waals (E_vdW_), the electrostatic (E_EL_) interactions, the polar (E_GB_) and nonpolar (E_SURF_) contributions. The van der Waals and the electrostatic interactions are the standard MM energy terms, and the polar term is obtained generally by using the generalized Born (GB) model, while the nonpolar is typically modeled with a term proportional to the solvent accessible surface area (SASA) [57]. Here, the python script MMPBSA.py in AMBER 16 was used in calculating the binding free energy and the per-residue energy decomposition for the hCa_v_1.3 and hCa_v_1.2 against the PYTs. A total of 1000 snapshots were extracted from the last 40 ns trajectory from the production simulation to calculate the MM/GBSA free energy. To exhibit the binding conformation between receptor and ligand, the best representative conformation of each complex was selected using a clustering algorithm [58]. Per-residue energy decomposition was also performed to evaluate the energy contribution of each residue in the systems. All the other parameters were kept as default values. VMD 1.9.2 [59], Pymol 1.8 [60] packages and Discovery Studio Client [61] were used to display an analysis of the MD trajectories and the binding conformations.

## 5. Conclusions

In this study, six PYTs were selected to study the selectivity for hCa_v_1.3 and hCa_v_1.2 through computational dynamic simulations. The results indicate that the hydrophobic surface formed by the residues located in IR2 and IR3 play an important role between PYTs and Ca_v_1.3/Ca_v_1.2. V162, L303, M481 and F484 residues (located in hCa_v_1.3) provided most of the energy contribution by the formation of hydrophobic interactions. The substituent of PYTs also affected the binding free energy. A polar group, generally, was not favorable. Suitable cycloalkyl and norbornyl groups could increase the selectivity to Ca_v_1.3. Moreover, a halogen-substituted aromatic ring helped PYTs to bind hCa_v_1.3/hCa_v_1.2. There are still some uncertain issues, such as the energy abnormality of PYT67, which need to be solved, so we will refine and improve the final results in a further study.

## Figures and Tables

**Table 1:** Binding free energy between hCav1.3/Cav1.2 and PYTs of group A.

Complexes	ΔE_v__d__W_	ΔE_EL_	ΔE_GB_	ΔE_SURF_	ΔE_total_
hCa_v_1.3-PYT06	−43.11	−3.85	18.06	−5.05	−33.95
hCa_v_1.2-PYT06	−28.73	−4.55	18.20	−3.68	−18.77
hCa_v_1.3-PYT22	−44.44	−4.56	18.95	−5.14	−35.19
hCa_v_1.2-PYT22	−37.14	−3.77	15.73	−4.48	−29.66
hCa_v_1.3-PYT65	−36.04	−5.34	19.27	−4.44	−26.56
hCa_v_1.2-PYT65	−32.76	−9.78	21.44	−4.25	−25.35

All the energies are in kcal/mol; ΔE_vdW_: van der Waals interaction energy; ΔE_EL_: electrostatic interaction energy; ΔE_GB_: polar solvation energy contribution; ΔE_SURF_: nonpolar solvation energy contribution; ΔE_total:_ the total binding free energy by MM/GBSA.

**Table 2:** Binding free energy between hCav1.3/Cav1.2 and PYTs of group B.

Complexes	ΔE_vdW_	ΔE_EL_	ΔE_GB_	ΔE_SURF_	ΔE_total_
hCa_v_1.3-PYT108	−38.49	−4.64	16.21	−5.09	−32.01
hCa_v_1.2-PYT108	−34.87	−2.84	18.25	−4.37	−23.84
hCa_v_1.3-PYT103	−42.42	−15.25	26.89	−5.11	−35.90
hCa_v_1.2-PYT103	−38.34	−2.00	13.78	−4.23	−30.78
hCa_v_1.3-PYT67	−32.77	−10.40	24.22	−4.07	−23.02
hCa_v_1.2-PYT67	−35.24	−5.16	18.60	−4.18	−25.99

All the energies are in kcal/mol; ΔE_vdW_: van der Waals interaction energy; ΔE_EL_: electrostatic interaction energy; ΔE_GB_: polar solvation energy contribution; ΔE_SURF_: nonpolar solvation energy contribution; ΔE_total_: the total binding free energy by MM/GBSA.

## Supplementary Materials

The following are available online, Figure S1: The sequence alignments of hCa_v_1.3 and hCa_v_1.2 with rCa_v_1.1, Figure S2: The structure of homologous models for hCa_v_1.3 and hCa_v_1.2, Figure S3: The Ramachandran plot of the models of hCa_v_1.3 and hCa_v_1.2, Figure S4: The homology models of hCa_v_1.3 and hCa_v_1.2 with lipid bilayer membranes and TIP3P water models, Table S1: The information of PYTs in the group A and group B for hCa_v_1.2 and hCa_v_1.3, Table S2: Evaluation results of models of hCa_v_1.3 and hCa_v_1.2, Table S3: The residues in major interactional regions of hCa_v_1.3 and hCa_v_1.2 complexes of group A, Table S4: The residues in major interactional regions of hCa_v_1.3 and hCa_v_1.2 complexes of group B.
