# Supplementary material for: Investigation of the Selectivity of L-Type Voltage-Gated Calcium Channels 1.3 for Pyrimidine-2,4,6-Triones Derivatives Based on Molecular Dynamics Simulation
Source: Molecules. 2020 Nov 20;25(22):5440. doi: 10.3390/molecules25225440 (PMC7699898; doi:10.3390/molecules25225440)
Supplement: Supplementary file 1 [file molecules-25-05440-s001.pdf]

molecules
MDPI

Supplementary Information

For the two groups, the PYTs of group A was selected for similar structure, the five-membered ring and the chlorophenyl were substituted on both sides of the mother nucleus of PYT. As for group B, we chose the PYTs with a large difference from group A structurally, especially the norbornyl in PYT103 and PYT108 might had an important effect on affinity. In view of these, the group A was used to explore the binding mode of PYT with $hCa_v1.2$ and $hCa_v1.3$, and group B was the parallel experiment to verify the reliability of the former.

Supplementary Table 1. The information of PYTs in the group A and group B for $hCa_v1.2$ and $hCa_v1.3$ [1, 2].

Group	PYTs	$hCa_v1.3$ IC_{50} (μM)	$hCa_v1.2$ IC_{50}(μM)	Selectivity[1]
	06	6.3	>100	>100
A	22	1.7±0.1	2.3±0.4	1.4
	65	>100	>100	-
	67	>100	>100	-
B	103	2.3±0.4	2.8±0.3	1.4
	108	2.1±0.1	76.6±0.8	36

[1]Ratio of the IC_{50} values ($hCa_v1.2$/$hCa_v1.3$).

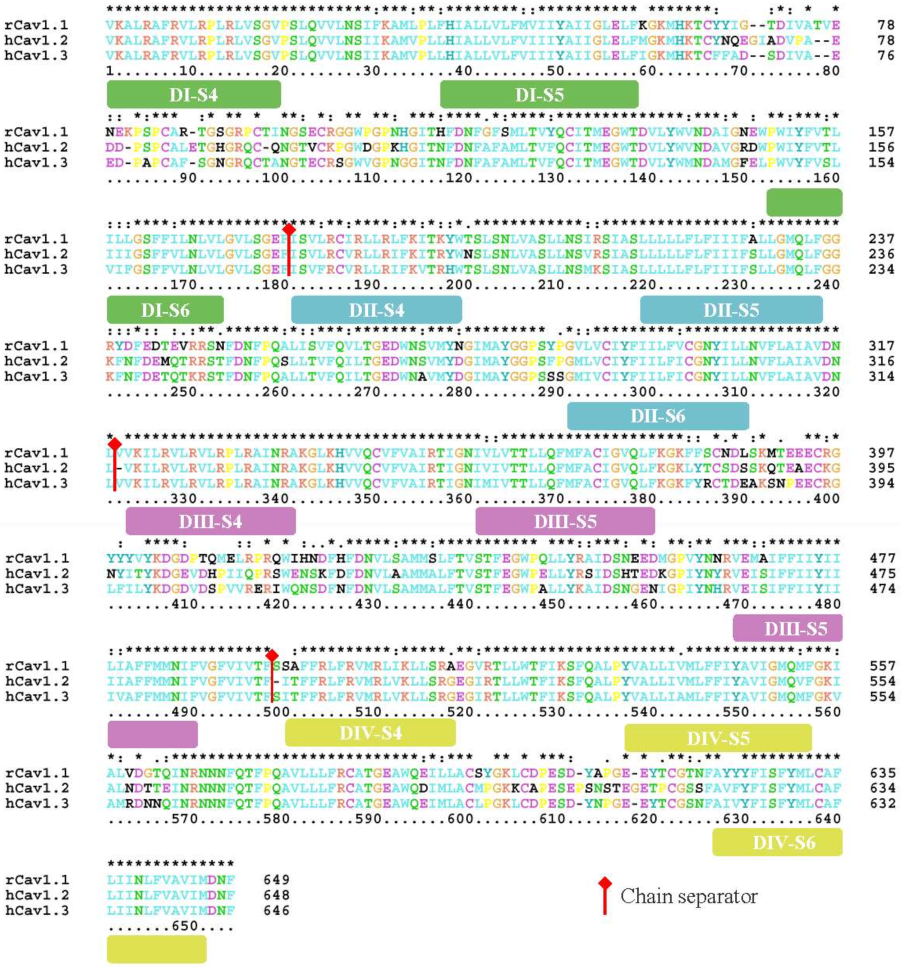

Supplementary Figure 1. The sequence alignments of hCa$_V$1.3and hCa$_V$1.2with rCa$_V$1.1.

Supplementary Table 2. Evaluationresults of models of hCa$_V$1.3 and hCa$_V$1.2.

Servers	Terms	hCa$_V$1.3	hCa$_V$1.2
ERRAT	Overall Quality Factor	73.89	70.75
PROCHECK (Ramachandran plot(%))	most favored regions	88.7	89.1
	additional allowed regions	8.8	9.4
	generously allowed regions	2.1	1.4
	disallowed regions	0.3	0.2
WHATCHECK	Bond Lengths RMSZ-score[1]	0.880	0.876
	Bond Angle RMSZ-score[1]	1.654	1.390

[1]RMS Z-score: The number of "standard deviations away from the mean" is called "Z", and RMS Z-scores mean the "root mean square" of a population of Z values. RMS Z-scores should be close to 1.0.

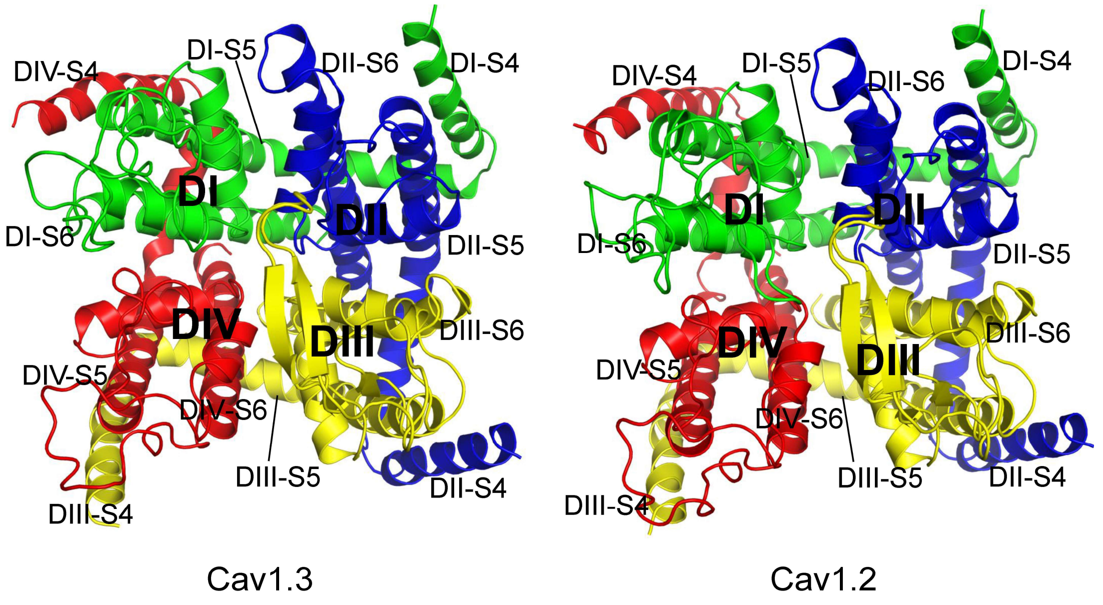

Supplementary Figure 2. The structure of homologous models for hCa$_V$1.3 and hCa$_V$1.2.The domain was markedbased on the names.

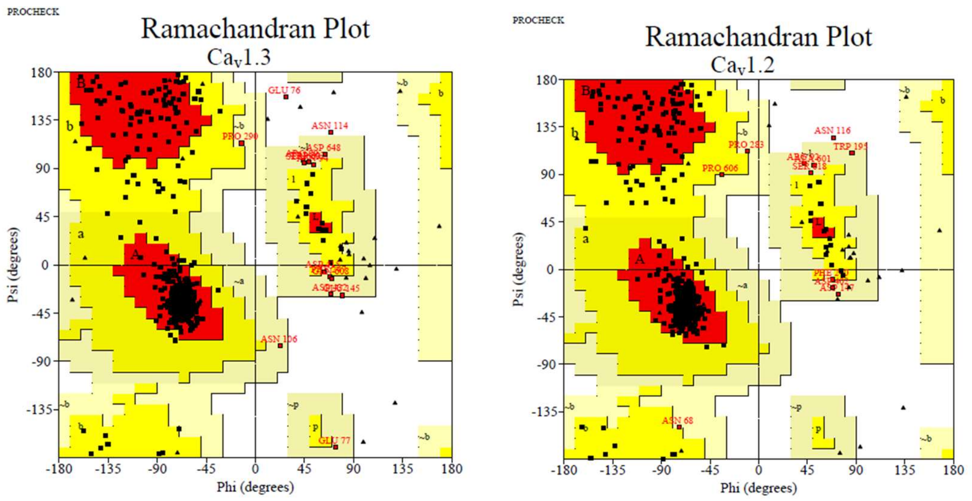

Supplementary Figure 3. The Ramachandran plot of the models of hCa$_v$1.3 and hCa$_v$1.2 .

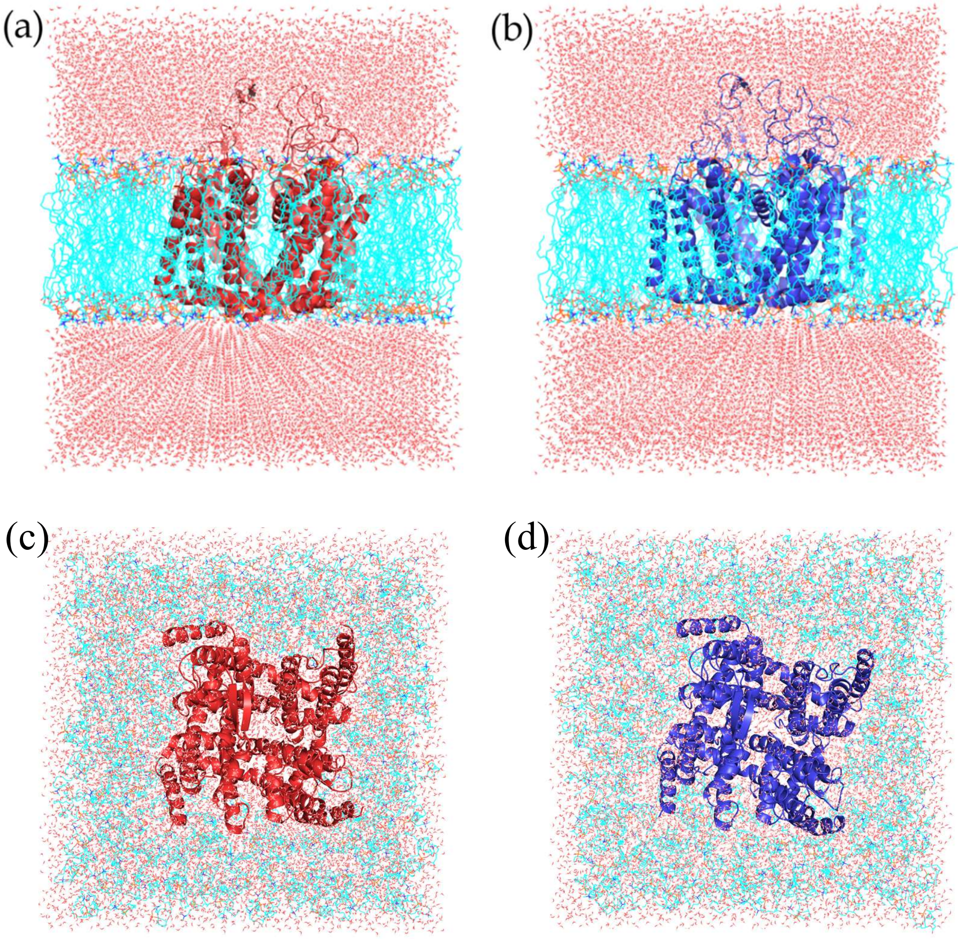

Supplementary Figure 4. The side view and top view of homology models of hCa$_v$1.3(a,c) and hCa$_v$1.2 (b,d) with lipid bilayer membranes and TIP3P water models.

Supplementary Table 3. The residues in major interactional regions of hCa$_v$1.3 and hCa$_v$1.2 complexes of group A.

Complexes	IR1	IR2	IR3	IR4
hCa$_v$1.3-PYT06	V162, L165	N300,L303,L304, F307	M481, F484	-
hCa$_v$1.2-PYT06	V164	L305	M481, F485	-
hCa$_v$1.3-PYT22	V162,163, V166	F307	T436,481, F484	I634
hCa$_v$1.2-PYT22	V164	L306	M481, F485	I637
hCa$_v$1.3-PYT65	V162	-	F484	C630,631, I634, F638
hCa$_v$1.2-PYT65	-	Q364	I477, M481, I484	I637

Supplementary Table 4. The residues in major interactional regions of hCa$_v$1.3 and hCa$_v$1.2 complexes of group B.

complexes	IR1	IR2	IR3	IR4
hCa$_v$1.3-PYT108	-	L303,	A477,M480, M481, F484,	-
hCa$_v$1.2-PYT108	I128, T129, V164	F298	-	-
hCa$_v$1.3-PYT103	V162, V166	L262, F296, N300, L303	F437, M481	-
hCa$_v$1.2-PYT103	L165	L305, L306	M481, M482, F485	-
hCa$_v$1.3-PYT67	V163	-	-	T627, C630, A631, I634
hCa$_v$1.2-PYT67	V164, V168	L305, L306, L309	M481,M482, F485	-

References

1. Kang, S.; Cooper, G.; Dunne, S. F.; Dusel, B.; Luan, C. H.; Surmeier, D. J.; Silverman, R. B., CaV1.3-selective L-type calcium channel antagonists as potential new therapeutics for Parkinson's disease. *Nat Commun* **2012,** *3*, 1146.
2. Kang, S.; Cooper, G.; Dunne, S. F.; Luan, C. H.; Surmeier, D. J.; Silverman, R. B., Structure-activity relationship of N,N'-disubstituted pyrimidinetriones as Ca(V)1.3 calcium channel-selective antagonists for Parkinson's disease. *J Med Chem* **2013,** *56* (11), 4786-97.
